# MarNemaFunDiv: a first comprehensive dataset of functional traits for marine nematodes

**DOI:** 10.1038/s41597-025-05105-6

**Published:** 2025-05-06

**Authors:** Edwin Daché, Daniela Zeppilli, Jozée Sarrazin, Ravail Singh, Elisa Baldrighi, Dmitry Miljutin, Aurélien Boyé

**Affiliations:** 1 Univ. Brest, Ifremer, UMR6197 Biologie et Ecologie des Ecosystèmes marins Profonds, F-29280 Plouzané, France; 2 National Oceanography Centre, Southampton, UK; 3 Department of Biology, University of Nevada-Reno, Reno, NV USA; 4 BioConsult GmbH & Co. KG, auf der Muggenburg 30 28217, Bremen, Germany; 5 IFREMER–DYNECO/LEBCO, Centre de Bretagne, CS1007 29280, Plouzané, France

**Keywords:** Ecosystem ecology, Biodiversity

## Abstract

Here, we present the first comprehensive dataset of functional traits for marine nematodes (MarNemaFunDiv). In this study, we propose 16 functional traits (life strategy, body shape, trophic group, oesophageal bulb, cuticle complexity, adhesive structures and ambulatory setae, head shape, amphid (shape and size), sensory structures (head and rest of the body), light sensing, male reproductive system (spicule, pre/postcloacal supplements and gubernaculum) and tail shape). Some of these traits were already used in marine ecology as functional categories (e.g. trophic groups, tail shapes, c-p classes) while others have never been considered before. These 16 traits were described and attributed to 86 nematode genera, representing the most abundant ones in shallow-water and deep-sea ecosystems. The matrix proposed in this study encompasses a comprehensive range of traits, enabling it to tackle a variety of ecological questions in the future.

## Background & Summary

The term meiofauna refers to a group of small benthic eukaryotic organisms and represents a fundamental and diverse component of marine ecosystems^1^. Meiofaunal metazoans are largely dominated by free-living nematodes, which play a crucial role in ecosystem processes and functions^2^. Nematodes are also used successfully as ecological indicators and sentinels for ecosystem health^3^. Despite their ecological significance, only a small fraction of their diversity has been described, and the taxonomic challenges remain particularly significant for this important benthic component. Due to their small size, the limited number of taxonomists, and the high percentage of undescribed species, taxonomic impediments severely constrain the use of meiofauna in ecosystem management.

Functional trait-based approaches offer a promising foundation for building integrated frameworks that bridge ecological theory and empirical evidence across multiple scales^4^. This approach bypasses the need for taxonomic expertise, enabling the understanding of ecological dynamics along environmental gradients based on species function rather than their taxonomy^5^. In recent years, the use of trait-based approaches has increased in marine ecology due to their potential in addressing macroecological questions, including ecosystem functioning^6^. The growth of trait-based approaches has been facilitated by the expanding availability of trait databases covering a wide array of taxa and ecosystems (see Martini *et al*.^4^ for a comprehensive review of existing databases). However, the absence of such databases for marine nematodes has hindered the application of this approach to this essential component of biodiversity.

Several studies have highlighted the relationship between nematode morphology and functions^7^. Taxonomic-based approaches allow to classify nematodes into different trophic and life history categories^8–11^. This functional approach has been proved effective for detecting environmental changes^12^, with trait-based indicators being particularly efficient for understanding the response of terrestrial nematodes^5^. For example, functional trait-based approaches have been shown to be more sensitive and reliable than taxonomy-based approaches in reflecting changes in soil nutrients^13^. While feeding groups and life history are widely applied in ecological studies of marine nematodes^14–30^, many other morpho-functional traits of free-living nematodes, linked to important ecological functions^30–42^, remain rarely investigated. For marine nematodes, the only morpho-functional traits possibly considered were tail and body shape^30,35,41^. Only a few studies proposed other morpho-functional traits such as the amphid shape^30–42^ or cuticle patterns^25,30,33–35,40,41,43–45^.

Although marine nematodes are valuable indicators of environmental change and anthropogenic impact, their use as ecological indicators remains limited, primarily due to taxonomic challenge. Trait-based approaches offer a promising methodology for integrating marine nematodes into macroecological studies, yet open-access datasets remain scarce and include only a limited number of functional traits. This study provides a freely accessible data matrix (MarNemaFunDiv) describing trait expressions of 86 marine nematode genera with a selection of 16 biological traits^46^. This paper also provides the description of selected traits and their respective modalities.

## Methods

### Selection of nematode genera

We selected the most abundant genera from contrasting shallow-water and deep-sea ecosystems. For shallow-water environments, we included nematodes from regular sandy beaches (Baldrighi *et al*.^47^), sites impacted by green algae blooms (Baldrighi *et al*.^47^), maerl beds (Rebecchi *et al*.^48^), anoxic sediments from a harbour and, shallow-water hydrothermal vents (Baldrighi *et al*.^49^). From deep-sea ecosystems, we selected nematodes from inactive sediments surrounding deep-sea around hydrothermal vents (Spedicato *et al*.^50^), polymetallic nodules fields (Miljutina *et al*.^51^), pockmarks (Sanchez *et al*.^52^) and seamounts (Zeppilli *et al*.^53^).

All nematode genera reported in these studies were identified at genus level. For the last dataset (nematodes from anoxic sediments from the Roscoff harbour; unpublished data), specimens were identified by the authors (identification made on a microscope on nematodes mounted on slides following the formalin–ethanol–glycerin protocol of De Grisse 1969^54^ and according to Platt & Warwick, 1983, 1988; Warwick, Platt & Somerfield, 1998; Schmidt-Rhaesa, 2014^55–58^). For each study, we considered the most abundant genera and selected only those representing more than 5% of the total nematode community. Therefore, 86 genera (Supplementary Table S1) were considered for this dataset.

### Selection of traits

**Fig. 1:**
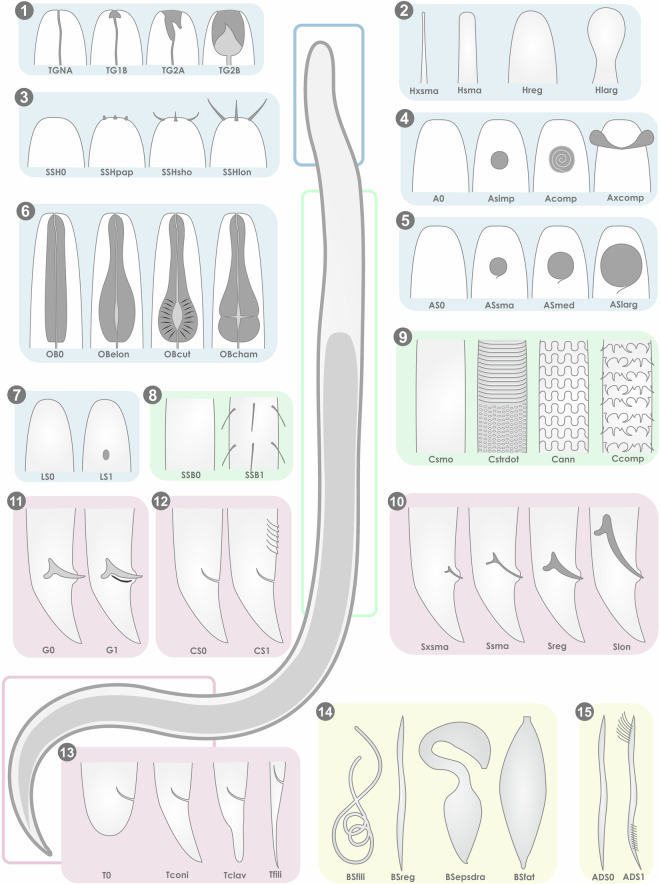
Schematic illustration of functional traits defined for meiofaunal Nematodes. (1) Trophic group: non actif and selective deposit feeders (TGNA), non-selective deposit feeders (TG1B), epigrowth and epistrate feeders (TG2A), predator and omnivores and facultative predators (TG2B); (2) Head shape: very small and neck elongated (Hxsma), small normal neck (Hsma), normal (Hreg), larger than neck and capsuled and helmet (Hlarg); (3) Sensory structures of head: absence (SSH0), papilliform (SSHpap), short setae (SSHsho), long setae (SSHlon); (4) Amphid shape: absence (A0), simple (Asimp), complex (Acomp), very complex and external amphid (Axcomp); (5) Amphid size: absence (AS0), with a diameter <30% of the head diameter (ASsma), with a diameter between 30% and 60% of the head diameter (ASmed), with a diameter >60% of the head diameter (ASlarg); (6) Oesophagal bulb: absence (OB0), elongated (OBelon), cuticularized (OBcut), with chambers (OBcham); (7) Light sensing: absence (LS0), presence (LS1); (8) Sensory structure rest of the body: absence (SSB0), presence (SSB1); (9) Cuticle: smooth (Csmo), striated and dotted (Cstrdot), annulated and strong cuticular pattern (Cann), complex structures (Ccomp); (10) Spicule: very small (Sxsma), small and thin (Ssma), normal (Sreg), complex and very long (Slon); (11) Gubernaculum: absence (G0), presence (G1); (12) Pre/Postcloacal supplementary organs: absence (CS0), presence (CS1); (13) Tail shape: absent and truncated and swollen (T0), conical (Tconi), clavate (Tclav), filiform and very long (Tfili); (14) Body shape: filiform (BSfili), regular (BSreg), epsilon and draco (BSepsdra), fat and large (BSfat); (15) Adhesive structure and/or ambulatory setae: absence (ADS0), presence (ADS1). The functional trait ‘Life Strategy’ is not represented in this figure.

Nematodes exhibit diverse morphologies, with variations in physiology and life history strategies that influence their development, reproduction, and survival in response to environmental changes^5^. Quantitative morphological traits, such as body size, are often considered “master” traits due to their significant ecological implications (e.g., Martini *et al*.^4^). However, in this study we chose to exclude body size and other morphometric traits which usually express high variability within genera, making it challenging to represent a single, consistent value for each genus. An example of this variability is seen in *Sabatieria* nematodes, whose body sizes can span a broad range from a few millimetres to much larger forms highlighting the difficulty of assigning a single representative value^58–61^. Including such a variable trait might introduce inconsistency or confusion for users of this dataset. The current focus of the dataset is on traits that are relatively stable and comparable across genera, allowing for standardized functional categorization. Adding body size, or other variable morphometric traits, would require defining ranges or averages, which might not adequately represent the ecological diversity within each genus. For this study, we selected traits possibly reflecting their responses to environmental changes (response traits) or proxies of their influence on ecosystem functions (effect traits). Some of these traits are already widely used in marine ecology as functional categories (e.g. trophic groups, tail shapes, c-p classes), while others have never been considered before. A total of 16 biological traits were defined (Fig. 1) including morphological traits such as body shape, buccal cavity structure, oesophageal bulb, cuticle complexity, adhesive structures and ambulatory setae, head shape, amphid shape and size, light sensing, male reproductive system (spicule, supplementary organs and gubernaculum), tail shape as well as life history traits such as life strategy (Table 1; Table 2). These 16 traits were divided into 58 modalities, with 5 traits being binary (presence/absence) and the remaining divided into up to 4 modalities. The proposed trait modalities follow the taxonomic descriptions of Platt and Warwick of 1983^55^ and *Handbook of zoology volume 2 Nematoda* Edited by: Andreas Schmidt-Rhaesa of 2014^58^.

**Table 1:** Codes associated with different modalities and functional traits.

Functional Traits	Modality	Code
Life strategy	CP1	LSCP1
	CP2-3	LSCP2
	CP4	LSCP3
	CP5	LSCP4
Body shape	Filiform	BSfili
	Regular	BSreg
	Epsilon-Draco	BSepsdra
	Fat-Large	BSfat
Trophic group	Non active (Symbiose/Reserve) +1 A (Selective deposit feeders)	TGNA
	1B (Non-selective deposit feeders)	TG1B
	2 A (Epigrowth/Epistrate feeders)	TG2A
	2B (Predators and omnivores)	TG2B
Oesophagal bulb	Absence	OB0
	Elongated	OBelon
	Cuticularized	OBcut
	With chambers	OBcham
Cuticle	Smooth	Csmo
	Striated-dotted	Cstrdot
	Annulated & Strong cuticular pattern	Cann
	Complex structures	Ccomp
Adeshive structures and/or Ambulatory setae	Abscence	ADS0
	Presence	ADS1
Head	Very small, neck elongated	Hxsma
	Small normal neck	Hsma
	Normal	Hreg
	Larger than neck/Capsuled	Hlarg
Amphid	Absence	A0
	Simple	Asimp
	Complex	Acomp
	Very complex/External	Axcomp
Amphid size	Absence	AS0
	<30%	ASsma
	30-60%	ASmed
	>60%	ASlarg
Sensory structures of the head	Absence	SSH0
	Papilliform	SSHpap
	Short setae	SSHsho
	Long setae	SSHlon
Sensory structures rest of the body	Absence	SSB0
	Presence	SSB1
Light sensing	Absence	LS0
	Presence	LS1
Spicule	Very small	Sxsma
	Small thin	Ssma
	Normal	Sreg
	Complex/Very long	Slon
Pre/postcloacal suppl	Absence	CS0
	Presence	CS1
Gubernaculum	Absence	G0
	Presence	G1
Tail	Absence/Truncated/Swollen	T0
	Conical	Tconi
	Clavate	Tclav
	Filiform/Very long	Tfili

**Table 2:** Categorization of nematode traits selected in this study with their ecological and functional relevance.

	Trait	Modalities	Functional and ecological relevance
*Life history traits*	Life strategy	Extreme colonisers (c-p 1), intermediate with increasing in abundance in stressed or eutrophic conditions (c-p 2-3), sensitive to stress (c-p 4), extreme persisters (c-p 5).	Evolutionary adaptions to disturbed environments
*Morphological traits*	Body shape	filiform, regular, S-shape, swollen	Mobility, energy requirements, oxygen requirements, capability to cope with stress
	Trophic group	non active (symbiosis) and selective deposit feeders, non-selective deposit feeders, epigrowth/epistrate feeders, predators/omnivores/facultative predators	Feeding habits
	Oesophageal bulb	absent, elongated, cuticularized, with chambers	Feeding efficiency
	Cuticle morphology	smooth, striated-dotted, with annulated and strong cuticular patterns with complex structures	Defence capacity and mobility. Relation to hydrodynamic and grain size.
	Adhesive structures and ambulatory setae	Presence or absence	Mobility and anchoring
	Head shape	very small with neck elongated, small with normal neck, normal, larger than neck/capsuled/helmet	Feeding, mobility, defence capability
	Amphid shape and size	Shape: absence, simple, complex, very complex/external. Size: absence of amphid, amphid with a diameter <30% of the head diameter, amphid with a diameter between 30% and 60% of the head diameter, amphid with a diameter >60% of the head diameter	Feeding, reproduction, capability to cope with stress.
	Cephalic and peripheral sensory structures	Cephalic sensory structures: absence, papilliform sensory structures, short setae and long setae. Peripheral sensory structures: presence or absence	Feeding, reproduction, capability to cope with stress
	Light sensing	Presence or absence	Feeding, mobility
	Male reproductive system	Size of the spicule: very small, small and thin, normal, complex/very long. Gubernaculum: presence or absence Genital supplementary organs: presence or absence	Reproduction
	Tail shape	absent/truncated/swollen, conical, clavate, filiform/very long	Feeding, mobility, reproduction. Relation to sediment chemistry and grain size.

### Definition of traits and modalities

#### Life strategy

Bongers *et al*. (1991, 1995)^8,10^ classified nematode life history based on their colonization success rates. They proposed a five-point scale ranging from extreme r–strategists or colonizers (characterized by short generation times, high reproduction rates, high colonization abilities, tolerant to disturbances, high metabolic activity and opportunistic behavior) to k–strategists or persisters (i.e., with long life spans, low colonization abilities, few offspring, sensitive to disturbances, low metabolic activity, and later appearance in successional processes). When a genus was not listed in the Bongers’ classification, we assigned the family c-p score, considering that, in absence of other available information, life history is usually quite substantial at family level.

Modalities within life strategy have been adapted from Bongers *et al*. (1991, 1995)^8,10^, and include the following categories: extreme colonisers (c-p 1), intermediate with increasing in abundance in stressed or eutrophic conditions (c-p 2-3), sensitive to stress (c-p 4), extreme persisters (c-p 5) (Table 1).

#### Body shape

Nematodes exhibit a wide range of body shapes, from highly elongated, filiform forms to more swollen morphologies. These variations, which reflect adaptations to different sedimentary conditions, may influence nematode locomotion, energy demands, and stress tolerance. Body shape and size play crucial roles in various functional aspects, including life history, physiology, ecology, and energetic requirements^62–66^. Several studies have shown a link between resource distribution, carbon and nitrogen cycles and nematode body shapes and sizes^13,67,68^. In particular, nematode length can influence metabolic rates, stress tolerance, movement capacity, and defense against predation^62,69–71^. Shorter, slender nematodes are often associated with oligotrophic conditions, as their elongated bodies facilitate greater epidermal oxygen uptake. Studies have shown that this elongated body shape in nematodes is linked to low oxygen levels, stressful conditions, and oligotrophic environments^72–74^. Slender bodies were also reported to be associated with silt/clay sediments^25^. Investigating the response of nematodes to anthropogenic contamination, Egres and coauthors (2019)^22^ showed a dominance of stout bodies following a disturbance. The families *Draconematidae* and *Epsilonematidae* family have unique S-shape body form. This specific shape enables a distinctive mode of locomotion on their ventral side, moving in a “hirudinean” manner^75^.

In the present dataset, 4 body shape modalities were retained: filiform, regular, S-shape and swollen (Table 1; Fig. 1).

#### Buccal cavity structure (trophic groups)

Feeding mode describes energy, carbon and nutrient dynamics within the soil and the sediment food web. The available classifications of nematodes in different trophic groups are proposed according to nematode buccal cavity structures. The first classification was proposed by Wieser’s in 1953^76^, which assign nematodes in: group 1 A (selective deposit feeders) for nematodes with mouth very minute or almost absent; 1B (non-selective deposit feeders) for nematodes with large mouth but without teeth or other structures; 2 A (epigrowth feeders) for nematodes able to scrape food by teeth or plates; and 2B (predators and omnivores) for nematodes with powerful armature of teeth^76^. In 1997, Moens and Vincx^77^, proposed to split the group 1 A into two sub-groups (ciliate feeders and microvorous) and to separate 2B in two different sub-groups: predators and facultative predators. Another study proposed totally different trophic groups including (i) deposit-feeders swallowers, feeding on bacteria and unicellular organisms; (ii) epistrate-feeders tear-and swallow feeders, feeding on bacteria, diatoms, and other algae; (iii) chewers predators on protozoa, and metazoans; and (iv) suction feeders, omnivores feeding on algae, fungi, vascular plants, animals, epidermal cells, and root hairs^78^. A recent study suggested that specific trophic guilds rather than trophic groups proposed by Wieser (1953)^76^ would be more appropriate to detect environmental changes^12^. Furthermore, isotopes analyses revealed that marine free-living nematodes are more opportunistic than expected and that they can adjust their diet based on the available resources than solely relying on their trophic guilds^79^. Despite these alternative perspectives, we chose to use the Wieser’s classification for the proposed dataset, as it remains the most widely used framework in marine ecology studies and the most comprehensive classification available in the literature for free-living marine nematodes. Additionally, since the marine nematode families *Stilbonematinae* and *Astomonematina* are well known to have symbiotic relationships with micro-organisms^80^, we included them within the selective deposit feeder group.

The trophic groups trait in this dataset follows Wieser’s (1953)^76^ classification with slight adaptations and includes the following modalities: non active (symbiosis) and selective deposit feeders, non-selective deposit feeders, epigrowth/epistrate feeders and predators/omnivores/facultative predators (Table 1; Fig. 1).

#### Oesophageal bulb

The nematode pharynx can be particularly complex, with the presence of a terminal bulb, also called oesophageal bulb, which helps propel food into the intestine through the action of strong musculature^58^. Small bacterivorous nematodes typically lack this bulb and instead possess a shorter, cylindrical pharynx^81^. The greater the number of cuticular linings and chambers in the bulb, the more efficient is the suction process^82^. To our knowledge, there is no information in literature linking the presence or morphology of the oesophageal bulb to specific environmental conditions. However, its presence is hypothesized to be associated with feeding efficiency.

In this study, this functional trait is categorized into four modalities: bulb absent, bulb elongated, bulb cuticularized, bulb with chambers (Table 1; Fig. 1).

#### Cuticle morphology

In free-living nematodes, the cuticle serves as a protective barrier between the organism and its surrounding environment^83^. Additionally, it functions as an exoskeleton, helping them to maintain their body shape and playing a critical role in locomotion^84^. In marine environments, the cuticle morphology and thickness can be influenced by sediment type and hydrodynamic conditions^85,86^. The cuticle provides protection against predators and helps nematodes cope with pollution^41,85,87,88^. Annulation in cuticles may facilitate locomotion and attachment, while ridges may enable the widening of the body^58^. Cuticular ornamentations help nematodes maintain a stable position in the surface sediment layers, and spines can also function as a scraping mechanism^58^. To our knowledge, only a few studies have proposed functional categories for the cuticle for marine nematodes^22,25,30,35,41,44^. Semprucci and co-authors (2018)^41^ categorized nematode cuticles into six types: (i) smooth; (ii) with desmens; (iii) with a bacteria covering; (iv) punctuated or annulated with or without lateral differentiation; (v) punctuated or annulated with longitudinal structures for the whole-body length; and (vi) with wide body annules and longitudinal ridges. Nematodes with ornamented cuticles have been found in impacted areas near an oil refinery by Egres *et al*.^22^. Additionally, ornamented cuticles were associated with sandy sediments in physically harsh estuarine environments^25^. Kalogeropoulou *et al*.^35^ observed that nematodes with smooth cuticles were completely absent in sites with extreme conditions. Justino *et al*.^30^ reported a significant relationship between cuticle characteristics and pollutant exposure.

For this study, the cuticle trait modalities have been adapted from Semprucci *et al*.^41^, and include: smooth cuticle, striated-dotted cuticle, cuticle with annulated and strong cuticular patterns and cuticle with complex structures (Table 1; Fig. 1).

#### Adhesive structures and ambulatory setae

Some nematodes possess adhesive structures and ambulatory setae^58^. In some nematodes ambulatory setae can be positioned on the ventral side of the posterior body, while in Draconematidae, adhesion tubes are positioned both on the head and on the ventral part of the body^89,90^. These tube-like structures allow nematodes to ambulate on surfaces, adhere to a substrate, or crawl over it in a manner similar to that of a geometrid caterpillar.

Modalities within this trait have been categorized into presence or absence of adhesive structures and/or ambulatory setae (Table 1; Fig. 1).

#### Head shape

Free-living marine nematodes are characterized by different head shapes, ranging from minute to larger and sclerotized head regions (helmet or capsule^58^). To our knowledge, there is no information in literature about functional relevance of the head shape in relation to the surrounding environments. We can hypothesize that the sensory systems present on the head may vary significantly between shape, affecting nematode chemical detection. Furthermore, locomotion and feeding can be impacted by the head size and shape.

Modalities within this trait have been categorized as very small head with an elongated neck, small head with normal neck, normal head and head larger than neck/capsuled/helmet (Table 1; Fig. 1).

#### Amphid (shape and size)

Amphids are the main and complex multifunctional sensory organs of nematodes, located in the cephalic region^58^. The distal part of the amphid, the fovea, is an excavation or invagination in the cephalic cuticle that forms a pocket. This special sensilla has olfactory, chemoreceptive, and thermoreceptive functions^91^ used for reproduction and feeding^41^. There is also evidence that amphids can have photoreceptive and secretory functions and can be sensitive to pH and ions^82^. Small amphids are typical of terrestrial nematodes living in environments rich in food resources, while large amphids are characteristic of nematodes from freshwater oligotrophic environments^92^. In marine ecosystems, only a few studies have explored the relationship between amphids and environmental conditions^35,41,44^. While Kalogeropoulou did not find any significant relationship between amphids and environmental conditions, rounded and elongate loops were found in nematodes from highly hydrodynamic environments^41^. Justino *et al*.^30^ reported a significant relationship between amphid fovea and pollutants.

In this study, amphid shapes have been categorized into the following modalities: absence of amphid, simple amphid, complex amphid, very complex/external amphid. Regarding the amphid size trait, the proposed modalities are absence of amphid, amphid with a diameter <30% of the head diameter, amphid with a diameter between 30% and 60% of the head diameter, and amphid with a diameter >60% of the head diameter (Table 1; Fig. 1).

#### Sensory structures (head and rest of the body)

Nematodes possess a complex diversity of sensory receptors that allow them to respond to a wide range of physical and chemical stimuli^93^. Their head carries several sensory structures, including mechano- and chemoreceptors. In particular, cephalic and labial sense organs can take the form of papilliform receptors with short or long setae. Additional sensory structures (primarily sensilla) may also be found on other parts of the nematode’s body. These sensilla can be numerous and arranged in dorsal, ventral and sublateral rows along the body. Dorsally, they are often restricted to the anterior neck region^82^. The function of these sensory structures is mainly tactile, but some setae may possess a tip opening suggesting a potential chemosensitivity role^93^. To our knowledge, the functional relevance of nematode sensory structures in relation with environmental conditions has only been explored by Kalogeropoulou *et al*.^35^ with no significant patterns observed. We hypothesize that the presence of these mechano- and chemoceptors may influence their feeding, reproductive success and their ability to cope with stress.

In this study, cephalic sensory structures have been categorized into the following modalities absence of cephalic sensory structure, papilliform sensory structures, short setae, and long setae. The modalities for the peripheral sensory structures are presence or absence (Table 1; Fig. 1).

#### Light sensing

Free-living aquatic nematodes can possess pigment spots or ocelli^58^. These photoreceptors are responsible for negative phototaxis guiding movements into deeper layers/strata^94^. They can also play a role in the searching for food.

The proposed modalities for light sensing are presence or absence (Table 1; Fig. 1).

#### Male reproductive system (spicule, pre/postcloacal supplements and gubernaculum)

The copulatory apparatus of nematode male reproductive system consists of two cuticularized spicules and associated gubernaculum, which are controlled by protractor and retractor muscles. The shape of the spiculum facilitates the opening of the vulva, allowing sperm to flow into the female. Each spiculum contains sensilla with receptors^58,82^. Some males may also possess genital supplementary organs (such as supplements, papillae and genital setae) with mechanoreceptive and secretory functions^58^, which can influence the reproduction success.

Modalities for spicule size have been categorized into very small spicules, small and thin spicules, normal spicules, complex/very long spicules. For gubernaculum and genital supplementary organs, the trait modalities selected are presence/absence (Table 1; Fig. 1).

#### Tail shape

Tail shape has been shown to play a role in locomotion, feeding and reproduction^41^. Thistle and Sherman (1985)^95^ proposed to dividing tails into 11 functional categories. This initial division was reduced to four categories by Thistle and co-authors (1995)^96^ and adopted by subsequent studies. In the marine domain, limited studies linking tail shapes to the environment revealed a relationship between clavate, conical and cylindrical tails and intermediate energy level conditions^41^, primarily influenced by salinity, oxygen and chlorophyll *a*^18^. In deep sea chemosynthetic environments, a higher diversity of tail shapes is observed compared to other types of deep-sea habitats, where elongated or filiform tails usually dominate^35^. Clavate tail shapes may be associated with a higher fraction of silt and clay in the sediment^25^, while elongate/filiform tails have been reported in fine sand and muddy sediments^22^. Filiform tail shapes can be also associated with a hemisessile lifestyle^96^.

Modalities for the trait tail shape have been adapted from Semprucci *et al*.^41^, and include four categories: tail absent, truncated, or swollen; conical tail; clavate tail; and filiform, very long (Table 1; Fig. 1).

### Trait expression

To describe trait expression, modality affinities were selected based on taxonomical expertise. For all traits, the taxa affinity to the trait modalities was one-hot encoded, i.e. since the modalities are mutually exclusive, a taxon that shows an affinity for a given modality of a trait (coded 1) will not exhibit an affinity for the other modalities (all coded 0). Indeed, most of the proposed traits include variation among species and can be considered specific genus, or they may vary only rarely within the same genus. These traits include: body shape, buccal cavity structure, oesophageal bulb, cuticle morphology, adhesive structures and ambulatory setae, head shape, amphid shape and size, cephalic and peripheral sensory structures, light sensing, male reproductive system and life strategy. For other traits, such as tail shape, some variations may be present within the same genus. In these cases, we carefully examined all species of the genus and selected the most prevalent modality. For example, the genus *Oncholaimus* comprises more than 150 species. Most of species within this genus have a clavate tail, while only a few have a conical tail. Given the very low percentage of representation for this modality, we suggest the clavate tail modality for the genus *Oncholaimus*.

## Data Records

A matrix of biological traits information for 86 marine nematode genera, resulting from methods described above, has been publicly deposited in Zenodo^46^. The taxonomic nomenclature in this dataset was obtained from the World Register of Marine Species^60^ (WoRMS; http://www.marinespecies.org) on the 01/06/2024.

## Technical Validation

We developed a comprehensive and detailed database of nematode functional traits. We obtained information about traits from original research literature, followed by secondary literature such as textbooks^8,10,12,13,18,22,25,30,35,41,44,55,58,62–96^. We gave precedence to literature from marine nematodes; when such sources were unavailable, we included literature on freshwater, terrestrial, and parasitic nematodes. To describe trait expression, modality affinities were selected based on literature, which includes key identification guides^55–57^ and authoritative online resources (Nemys^61^ https://www.nemys.ugent.be/ and WORMS^60^ https://www.marinespecies.org/). During the data collection process, we used the most up-to-date species names and accurate taxonomic keys. Users should be aware that some taxa exhibit multiple modes of expression (categories) for a single trait. In this study, we selected the mode most commonly expressed for each genus. For example, the majority of species in the genus *Oncholaimus* have a clavate tail. However, a few of them (3 on 155 species) show very short tail^97–99^. This does not imply that all modes occur with equal probability across different environments, as trait expression can be influenced by abiotic and biotic factors. For instance, a genus may modify its feeding mode in response to anthropogenic disturbance, hydrodynamic conditions, water temperature and chemistry, or interspecific interactions (e.g.). Because reliable data on the specific conditions driving trait expression are often lacking, this information was not incorporated into the trait matrix to avoid introducing spurious variability. While this is a common limitation of biological trait analysis (BTA), even for well-studied organisms^4^, users should carefully evaluate the reliability of BTA outputs in relation to their ecological questions. Nevertheless, this database provides a foundational reference, identifying the most commonly expressed trait modalities for each taxon. Aware of the variability of some traits, in the future is recommended to add quantitative ranges to remove bias and reveal the real functional variability of different species within a genus. As ecological knowledge advances, it can serve as a basis for developing more refined and nuanced BTA approaches in the future.

## Usage Notes

The matrix proposed in this study covers an inclusive set of traits. Users of this dataset should ponder that, due to the plasticity of trait expression within a genus, a single trait may exhibit multiple modes of expression (categories). For example, within the genus *Sabatieria*, body shape, can vary drastically from BSreg (*Sabatieria pulchra*) to BSfili (*Sabatieria longispinosa)*. In this study, we assigned the most commonly expressed trait within each genus. While this approach does not capture the full variability of morphometric traits, it may be more suitable for representing the ecological diversity within each genus. Trait expression may be influenced by abiotic or biotic pressures. For example, a species may alter its feeding mode in response to environmental stress^79^, which may affect the consistency of BTA outputs depending on the research questions posed^100^. Finally, we state that the associated trait dataset represents the best information available to the authors at the time of manuscript submission. New traits or species can be added to the dataset by providing this information, along with references, to the corresponding author. We also encourage users to review, validate and, if necessary, modify the trait information provided here before to use.

## Supplementary information

Supplementary Table 1

## Data Availability

No custom code was used to generate or process the data described in the manuscript.
