# Supplementary material for: MarNemaFunDiv: a first comprehensive dataset of functional traits for marine nematodes
Source: Sci Data. 2025 May 6;12:752. doi: 10.1038/s41597-025-05105-6 (PMC12056057; doi:10.1038/s41597-025-05105-6)
Supplement: Supplementary file 1 — Supplementary Table 1 [file 41597_2025_5105_MOESM1_ESM.docx]

| **Class** | **Order** | **Family** | **Genus** |
| --- | --- | --- | --- |
| *Chromadorea* | *Araeolaimida* | *Axonolaimidae* | *Odontophora* |
| *Chromadorea* | *Araeolaimida* | *Comesomatidae* | *Comesomoides* |
| *Chromadorea* | *Chromadorida* | *Chromadoridae* | *Chromadorella* |
| *Chromadorea* | *Chromadorida* | *Chromadoridae* | *Chromadorita* |
| *Chromadorea* | *Chromadorida* | *Chromadoridae* | *Endeolophos* |
| *Chromadorea* | *Chromadorida* | *Chromadoridae* | *Innocuonema* |
| *Chromadorea* | *Chromadorida* | *Chromadoridae* | *Neochromadora* |
| *Chromadorea* | *Chromadorida* | *Chromadoridae* | *Spilophorella* |
| *Chromadorea* | *Chromadorida* | *Chromadoridae* | *Karkinochromadora* |
| *Chromadorea* | *Chromadorida* | *Cyatholaimidae* | *Marylynnia* |
| *Chromadorea* | *Desmodorida* | *Desmodoridae* | *Chromaspirina* |
| *Chromadorea* | *Desmodorida* | *Desmodoridae* | *Desmodora* |
| *Chromadorea* | *Desmodorida* | *Desmodoridae* | *Metachromadora* |
| *Chromadorea* | *Desmodorida* | *Desmodoridae* | *Molgolaimus* |
| *Chromadorea* | *Desmodorida* | *Desmodoridae* | *Paradesmodora* |
| *Chromadorea* | *Desmodorida* | *Desmodoridae* | *Perspiria* |
| *Chromadorea* | *Desmodorida* | *Draconematidae* | *Apenodraconema* |
| *Chromadorea* | *Desmodorida* | *Draconematidae* | *Dracograllus* |
| *Chromadorea* | *Desmodorida* | *Epsilonematidae* | *Akanthepsilonema* |
| *Chromadorea* | *Desmodorida* | *Epsilonematidae* | *Epsilonema* |
| *Chromadorea* | *Desmodorida* | *Epsilonematidae* | *Glochinema* |
| *Chromadorea* | *Desmodorida* | *Epsilonematidae* | *Metaglochinema* |
| *Chromadorea* | *Desmodorida* | *Epsilonematidae* | *Metepsilonema* |
| *Chromadorea* | *Desmodorida* | *Epsilonematidae* | *Perepsilonema* |
| *Chromadorea* | *Desmodorida* | *Richtersiidae* | *Richtersia* |
| *Chromadorea* | *Desmodorida* | *Desmodoridae* | *Pseudochromadora* |
| *Chromadorea* | *Desmodorida* | *Desmodoridae* | *Spirinia* |
| *Chromadorea* | *Desmoscolecida* | *Desmoscolecidae* | *Desmoscolex* |
| *Chromadorea* | *Desmoscolecida* | *Desmoscolecidae* | *Greeffiella* |
| *Chromadorea* | *Desmoscolecida* | *Desmoscolecidae* | *Tricoma* |
| *Chromadorea* | *Monhysterida* | *Linhomoeidae* | *Disconema* |
| *Chromadorea* | *Monhysterida* | *Linhomoeidae* | *Linhomoeus* |
| *Chromadorea* | *Monhysterida* | *Linhomoeidae* | *Metalinhomoeus* |
| *Chromadorea* | *Monhysterida* | *Monhysteridae* | *Halomonhystera* |
| *Chromadorea* | *Monhysterida* | *Sphaerolaimidae* | *Doliolaimus* |
| *Chromadorea* | *Monhysterida* | *Xyalidae* | *Amphimonhystrella* |
| *Chromadorea* | *Monhysterida* | *Xyalidae* | *Cobbia* |
| *Chromadorea* | *Monhysterida* | *Xyalidae* | *Daptonema* |
| *Chromadorea* | *Monhysterida* | *Xyalidae* | *Elzalia* |
| *Chromadorea* | *Monhysterida* | *Xyalidae* | *Linhystera* |
| *Chromadorea* | *Monhysterida* | *Xyalidae* | *Manganonema* |
| *Chromadorea* | *Monhysterida* | *Xyalidae* | *Metadesmolaimus* |
| *Chromadorea* | *Monhysterida* | *Xyalidae* | *Omicronema* |
| *Chromadorea* | *Monhysterida* | *Xyalidae* | *Paramonohystera* |
| *Chromadorea* | *Monhysterida* | *Linhomoeidae* | *Terschellingia* |
| *Chromadorea* | *Monhysterida* | *Monhysteridae* | *Thalassomonhystera* |
| *Chromadorea* | *Monhysterida* | *Xyalidae* | *Xyala* |
| *Chromadorea* | *Plectida* | *Camacolaimidae* | *Diodontolaimus* |
| *Chromadorea* | *Plectida* | *Ceramonematidae* | *Pselionema* |
| *Chromadorea* | *Plectida* | *Leptolaimidae* | *Leptolaimus* |
| *Chromadorea* | *Araeolaimida* | *Axonolaimidae* | *Axonolaimus* |
| *Chromadorea* | *Araeolaimida* | *Comesomatidae* | *Paracomesoma* |
| *Chromadorea* | *Araeolaimida* | *Comesomatidae* | *Sabatieria* |
| *Chromadorea* | *Araeolaimida* | *Comesomatidae* | *Setosabatieria* |
| *Chromadorea* | *Chromadorida* | *Chromadoridae* | *Acantholaimus* |
| *Chromadorea* | *Chromadorida* | *Chromadoridae* | *Actinonema* |
| *Chromadorea* | *Chromadorida* | *Chromadoridae* | *Euchromadora* |
| *Chromadorea* | *Chromadorida* | *Cyatholaimidae* | *Paracanthonchus* |
| *Chromadorea* | *Desmodorida* | *Draconematidae* | *Cephalochaetosoma* |
| *Chromadorea* | *Desmodorida* | *Draconematidae* | *Dinetia* |
| *Chromadorea* | *Desmodorida* | *Epsilonematidae* | *Leptepsilonema* |
| *Chromadorea* | *Desmodorida* | *Microlaimidae* | *Aponema* |
| *Chromadorea* | *Desmodorida* | *Microlaimidae* | *Calomicrolaimus* |
| *Chromadorea* | *Desmodorida* | *Microlaimidae* | *Microlaimus* |
| *Chromadorea* | *Desmodorida* | *Desmodoridae* | *Psammonema* |
| *Chromadorea* | *Desmoscolecida* | *Cyartonematidae* | *Cyartonema* |
| *Chromadorea* | *Desmoscolecida* | *Desmoscolecoidae* | *Calligyrus* |
| *Chromadorea* | *Monhysterida* | *Monhysteridae* | *Monhystera* |
| *Chromadorea* | *Monhysterida* | *Xyalidae* | *Marisalbinema* |
| *Chromadorea* | *Monhysterida* | *Xyalidae* | *Promonhystera* |
| *Chromadorea* | *Monhysterida* | *Xyalidae* | *Theristus* |
| *Chromadorea* | *Plectida* | *Camacolaimidae* | *Deontolaimus* |
| *Enoplea* | *Enoplida* | *Anticomidae* | *Odontanticoma* |
| *Enoplea* | *Enoplida* | *Ironidae* | *Thalassironus* |
| *Enoplea* | *Enoplida* | *Oxystominidae* | *Halalaimus* |
| *Enoplea* | *Enoplida* | *Oxystominidae* | *Litinium* |
| *Enoplea* | *Enoplida* | *Oxystominidae* | *Oxystomina* |
| *Enoplea* | *Enoplida* | *Phanodermatidae* | *Crenopharynx* |
| *Enoplea* | *Enoplida* | *Rhabdolaimidae* | *Syringolaimus* |
| *Enoplea* | *Enoplida* | *Thoracostomopsidae* | *Paramesacanthion* |
| *Enoplea* | *Enoplida* | *Thoracostomopsidae* | *Trileptium* |
| *Enoplea* | *Enoplida* | *Oncholaimidae* | *Viscosia* |
| *Enoplea* | *Enoplida* | *Enchelidiidae* | *Bathyeurystomina* |
| *Enoplea* | *Enoplida* | *Oncholaimidae* | *Metoncholaimus* |
| *Enoplea* | *Enoplida* | *Oncholaimidae* | *Oncholaimus* |
| *Enoplea* | *Enoplida* | *Oncholaimidae* | *Prooncholaimus* |

**Supplementary Table S1 Classification of selected genera**
